# Overexpression of tomato *SlNAC1* transcription factor alters fruit pigmentation and softening

**DOI:** 10.1186/s12870-014-0351-y

**Published:** 2014-12-10

**Authors:** Nana Ma, Hailong Feng, Xia Meng, Dong Li, Dongyue Yang, Changai Wu, Qingwei Meng

**Affiliations:** State Key Laboratory of Crop Biology, College of Life Science, Shandong Agricultural University, Daizong Street, Tai’an, 271018 Shandong P. R. China

**Keywords:** Abscisic acid, Ethylene, Fruit ripening, Gene expression, *SlNAC1*, Tomato

## Abstract

**Background:**

Fruit maturation and ripening are genetically regulated processes that involve a complex interplay of plant hormones, growth regulators and multiple biological and environmental factors. Tomato (*Solanum lycopersicum*) has been used as a model of biological and genetic studies on the regulation of specific ripening pathways, including ethylene, carotenoid and cell wall metabolism. This model has also been used to investigate the functions of upstream signalling and transcriptional regulators. Thus far, many ripening-associated transcription factors that influence fruit development and ripening have been reported. NAC transcription factors are plant specific and play important roles in many stages of plant growth and development, such as lateral root formation, secondary cell wall synthesis, and embryo, floral organ, vegetative organ and fruit development.

**Results:**

Tissue-specific analysis by quantitative real-time PCR showed that *SlNAC1* was highly accumulated in immature green fruits; the expression of *SlNAC1* increased with fruit ripening till to the highest level at 7 d after the breaker stage. The overexpression of *SlNAC1* resulted in reduced carotenoids by altering carotenoid pathway flux and decreasing ethylene synthesis mediated mainly by the reduced expression of ethylene biosynthetic genes of system-2, thus led to yellow or orange mature fruits. The results of yeast one-hybrid experiment demonstrated that SlNAC1 can interact with the regulatory regions of genes related lycopene and ethylene synthesis. These results also indicated that SlNAC1 inhibited fruit ripening by affecting ethylene synthesis and carotenoid accumulation in *SlNAC1* overexpression lines. In addition, the overexpression of *SlNAC1* reduced the firmness of the fruits and the thickness of the pericarp and produced more abscisic acid, resulting in the early softening of fruits. Hence, in *SlNAC1* overexpression lines, both ethylene-dependent and abscisic acid-dependent pathways are regulated by *SlNAC1* in fruit ripening regulatory network.

**Conclusions:**

*SlNAC1* had a broad influence on tomato fruit ripening and regulated *SlNAC1* overexpression tomato fruit ripening through both ethylene-dependent and abscisic acid-dependent pathways. Thus, this study provided new insights into the current model of tomato fruit ripening regulatory network.

**Electronic supplementary material:**

The online version of this article (doi:10.1186/s12870-014-0351-y) contains supplementary material, which is available to authorized users.

## Background

Fruit ripening is a genetically regulated process that involves numerous metabolic changes in colour, flavour, texture and aroma; these changes are controlled by endogenous hormonal and genetic regulators and external signals (temperature, light and hydration) [1]. This process has been stimulated using tomato as an excellent model of fleshy fruit development and ripening; tomato has been utilised as an excellent model because of several advantages, including well-characterised ripening mutants, small genome size, high-density genetic maps, short life cycle, efficient transient and stable transformation and complete genome sequence [2-4].

On the basis of the induction of respiration and ethylene at the onset of ripening, scholars categorised tomato as a climacteric fruit. Ripening in climacteric fruits can also be initiated by exposure to exogenous ethylene. Grierson [5] reviewed that ethylene induces ripening in climacteric fruits by using tomato as a model. Antisense genes are used to suppress the expression of *ACO1* and *ACS2*, which respectively encode 1-aminocyclopropane-1-carboxylic acid (ACC) oxidase (ACO) and ACC synthase (ACS); ACO and ACS are the major enzymes involved in ethylene biosynthesis. ACS is encoded in tomato by a multi-gene family with at least eight members and three other putative genes in the genome sequence [4]. ACO is encoded in tomato by a multi-gene family of at least four characterised ACOs and three other putative genes in the genome sequence [4]. Previous studies also characterised the effect of ethylene on gene expression during climacteric ripening [6-9]. Ethylene induces the expressions of *ACS2* and *ACS4*, which are important in tomato fruit ripening [10,11]. The upregulation of *ACS1A* and *ACS4* at ripening initiation produces ethylene, which induces *ACS2* and *ACS4* to mediate autocatalytic ethylene synthesis, a process typically observed in climacteric ripening. *ACS2* and *ACO1* control ethylene production in tomato fruits [12].

The plant hormone abscisic acid (ABA) not only regulates seed dormancy, plant growth and development, and responses to environmental stresses [13-15] but also displays a pattern of change similar to ethylene at late stages of fruit development [2,16]. Because the ABA content in ABA-deficient mutants was 75% lower than the normal level, both the plant and fruit did not show the normal growth observed in the wild type; the total fruit weight and average fruit weight in ABA-deficient mutant fruits were reduced compared with wild-type fruit, and the plant weight was 50% lower in the ABA-deficient plant than in the wild type, indicating that ABA was not only required for plant growth, but was also indispensable for fruit development and ripening [16]. In addition, application of exogenous ABA can increase the pigmentation and promoted ripening of sweet cherry fruits [17]. Exogenous ABA accelerates fruit ripening, and fluridone or NDGA treatment delays fruit ripening by ABA inhibition [18]. Sun et al. [19] reported that suppressed SlNCED1 by RNA interference resulted in reduced ABA accumulation in transgenic fruit, which led to down-regulation of genes encoding major cell wall catabolic enzymes. These reports demonstrate that ABA plays important roles in fruit ripening.

Genes involved in rare mutations that completely inhibit normal ripening have been identified; such advancement is considered as a major breakthrough in determining the transcriptional control of tomato ripening [20]. These mutations include *rin* (ripening inhibitor), *nor* (non-ripening) and *Cnr* (colourless non-ripening). Gene cloning efforts have shown that *rin* results from the deletion of the last exon of a tomato MADS-box transcription factor gene (*LeMADS-RIN*); *rin* is necessary to promote tomato fruit ripening [21]. The mutation of *rin* affects all of the involved ripening pathways; this finding supports the function of this gene as a master regulator of ripening [22]. Chromatin immunoprecipitation coupled with DNA microarray analysis and transcriptome analysis have been performed to identify 241 direct RIN target genes that contain a RIN binding site and exhibit RIN-dependent positive or negative regulation during fruit ripening [23]. The targets of *RIN* include known genes, such as *ACS2*, *ACS4*, *NR* (*Never ripe*), *E8*, *PG* (polygalacturonase), *TBG4* (galactanase 4), *EXP1* (expansin 1), *PSY1* (phytoene synthase 1), *NOR*, *CNR*, *TDR4*, *HB-1* and *RIN* itself [24-26]. Another study has revealed new targets, including bHLH (basic helix-loop-helix), NAC (NAM, ATAF1/ATAF2, CUC2), basic leucine zipper (bZIP) transcription factor (TF), zinc finger protein and *APETALA2a* [23].

In addition to *RIN*, *Cnr* and *NOR*, other TF genes, including *AGAMOUS-LIKE1*, *HD-ZIP HOMEOBOX PROTEIN-1*, *ETHYLENE RESPONSE FACTOR6*, *FRUITFULL1*, *FRUITFUL2*, *SlMADS1* and *APRR2-Like*, function in tomato fruit ripening [27-36]. *NOR*, a member of the NAC domain family, functions upstream of ethylene in the tomato fruit ripening cascade; *NOR* mutation leads to a non-ripening phenotype similar to that observed in *rin* [2]. *SlNAC4* positively regulates fruit ripening by affecting ethylene synthesis and carotenoid accumulation [37]. However, the mechanisms of action of the other NAC TFs involved in fruit ripening remain unknown.

*SlNAC1* interacts with tomato leaf curl virus replication accessory protein and enhances viral replication [38]. This gene is also involved in abiotic stress [39,40] and pathogen infection response [41]. In the present study, the transcripts of *SlNAC1* highly accumulated in fruit tissues and increased with fruit ripening. The overexpression of *SlNAC1* reduced the accumulation of total carotenoid and lycopene, ethylene production, fruit firmness and thickness of pericarp, but increased abscisic acid (ABA) contents. In transgenic fruits, genes involved in lycopene and ethylene biosynthesis were downregulated, whereas genes related to lutein, β-carotene and ABA synthesis as well as genes related to cell wall metabolism were upregulated. In addition, yeast one-hybrid assay results indicated that SlNAC1 interacted with *SlPSY1*, *SlACS2* and *SlACO1*. These results suggested that SlNAC1 regulated tomato fruit ripening via ethylene-dependent and ABA-dependent ripening pathways in *SlNAC1* overexpression lines.

## Results

### *SlNAC1* is involved in tomato fruit ripening

**Figure 1:**
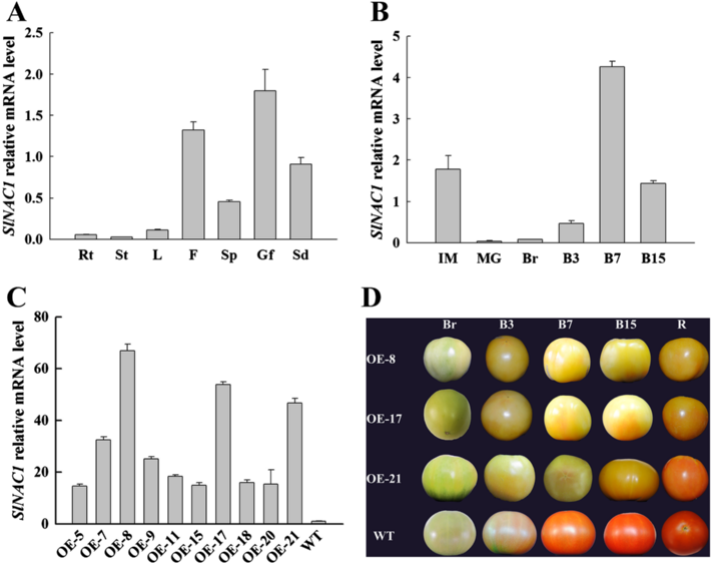
**qRT-PCR analysis of** ***SlNAC1*** **expression and phenotypes of OE and WT fruits. (A)** Transcripts of *SlNAC1* accumulated in different tissues. Rt, root; St, stem; L, leaf; F, flower; Sp, sepal; Gf, green fruit; Sd, seed. The pericarp tissues of the green fruits were used. **(B)** The relative mRNA level of *SlNAC1* as fruit ripened. The pericarp tissues of fruits at different stages were used to perform the experiment. **(C)** qRT-PCR expression analysis of *SlNAC1* in OE lines and WT. Total RNA from the pericarp tissues of fruits at B7 stage was subjected to quantitative RT-PCR analysis. **(D)** Phenotypes of OE and WT fruits along with the developmental stages. IM, immature green; MG, mature green; Br, breaker; B3, 3 d after breaker; B7, 7 d after breaker; B15, 15 d after breaker; R, ripe. Data are the means ± SD of three independent experiments. The WT expression data are normalised to 1.

The expression profile of *SlNAC1* in the roots, stems, leaves, flowers, sepals, green fruit and seeds was explored by quantitative real-time PCR (qRT-PCR). *SlNAC1* transcript showed higher expression levels in flowers, green fruits and seeds and the most transcripts of *SlNAC1* accumulated in green fruit, whereas lower expression levels were in root, stems and leaves (Figure 1A). These results suggested that *SlNAC1* may be related to fruit development. In line with this, transcripts of *SlNAC1* accumulated more at the immature green stage, then decreased at the mature green stage, and gradually increased until 7 d after breaker (Figure 1B). These results indicated that *SlNAC1* is likely to function in tomato fruit tissue.

To test whether *SlNAC1* is involved in tomato fruit ripening, we created the transgenic tomato lines by overexpressing this gene with its full-length cDNA under CaMV 35S promter. Ten independent overexpressed (OE) transgenic lines were obtained. Among them, OE-8, OE-17 and OE-21were detected with the higher expression levels of *SlNAC1* (Figure 1C), and were selected to perform the further experiments. In Figure 1D, the ripe fruits of three OE lines exhibited yellow or orange appearance compared with those of the wild-type (WT) line. These data indicate that overexpression of *SlNAC1* inhibits normal fruit ripening.

**Figure 2:**
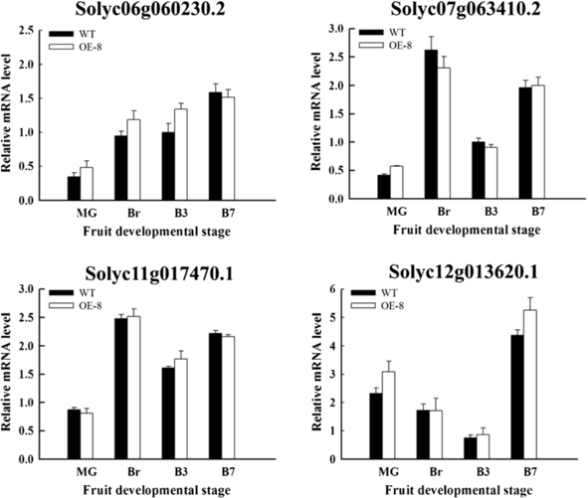
**Expression of four other NAC transcription factors in OE and WT fruits.** The pericarp tissues of fruits in different stages were used to perform the experiment. MG, mature green; Br, breaker; B3, 3 d after breaker; B7, 7 d after breaker. Data are the means ± SD of three independent experiments.

Approximately 101 NAC TFs in tomato are found in The Plant Transcription Factor Database (http://planttfdb.cbi.pku.edu.cn/) [42]. To confirm only *SlNAC1* was overexpressed in transgenic plants, we tested the expression of four other *NACs* that contain sequences with higher homology to *SlNAC1*, as indicated in the phylogenetic tree in the database. In Figure 2, the expression levels of these four other *NACs* were not significantly different at different stages between OE and WT fruits. These data indicated that overexression of *SlNAC1* does not affect the expression of closely related NAC genes.

### Overexpression of *SlNAC1* gene affects fruit colouring and related gene expressions

**Figure 3:**
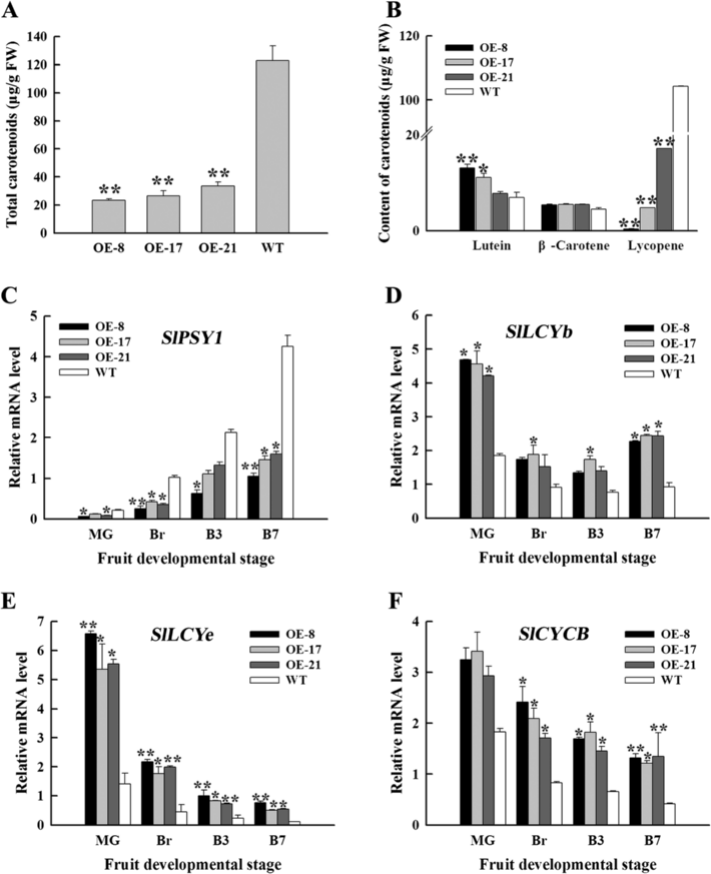
**Carotenoids contents and expression of carotenoid biosynthesis genes in OE and WT fruits. (A)** Total carotenoid content in OE and WT fruits at B20. **(B)** Contents of lutein, β-carotene and lycopene in OE and WT fruits at B20. **(C) to (F)** Expression analysis of genes related to carotenoid synthesis. The relative mRNA levels of *SlPSY1* **(C)**, *SlLCYb* **(D)**, *SlLCYe* **(E)** and *SlCYCB* **(F)** at indicated developmental stages were shown. MG, mature green; Br, breaker; B3, 3 d after breaker; B7, 7 d after breaker. Data are the means ± SD of three independent experiments. The asterisks indicate statistically significant differences between OE and WT fruits (*P < 0.05, **P < 0.01).

Lycopene is responsible for the red pigmentation of ripe tomato fruit; lycopene accounts for 70% to 90% of carotenoids in most varieties, whereas β-carotene accounts for the bulk of the remaining proportion (5% to 40%) [43,44]. In the present study, the total carotenoid contents of OE fruits were significantly reduced to 19.1%, 21.6% and 27.3% of WT fruits in OE-8, OE-17 and OE-21, respectively (Figure 3A). The lutein contents of OE fruits accumulated by 1.1 to 1.9 times higher than those of WT fruits and β-carotene contents of OE fruits also accumulated about 1.2 times higher than those of WT fruits. However, the lycopene contents in OE fruits were reduced by 83.5% to 99.6% compared with that of the WT fruits (Figure 3B). These data are consistent with fruit pigmentation.

Considering the distinct changes in the carotenoid composition of OE fruits, we analysed the genes’ transcript levels involved in carotenoid biosynthesis (Additional file 1: Figure S1) by qRT-PCR. PSY1 is a rate-limiting enzyme of carotenoid biosynthesis in tomato and is partly responsible for regulating flux via the pathway during ripening [45,46]. qRT-PCR results suggested that the expression level of *SlPSY1* was downregulated in the OE fruits accounting for reduced lycopene and total carotenoids in ripening fruits (Figure 3C). However, *SlLCYb* (lycopene β-cyclase), *SlLCYe* (lycopene ε-cyclase) and *SlCYCB* (chromoplast-specific lycopene β-cyclase) were upregulated in OE fruits, demonstrating the metabolism of lycopene to β-carotene and lutein (Figures 3D to 3F). These results suggest that the altered pigmentation of OE fruits is consistent with the changes in the expression of genes related to lycopene synthesis and decomposition observed.

### Overexpression of *SlNAC1* reduces ethylene emission by downregulating the genes related to ethylene biosynthesis

**Figure 4:**
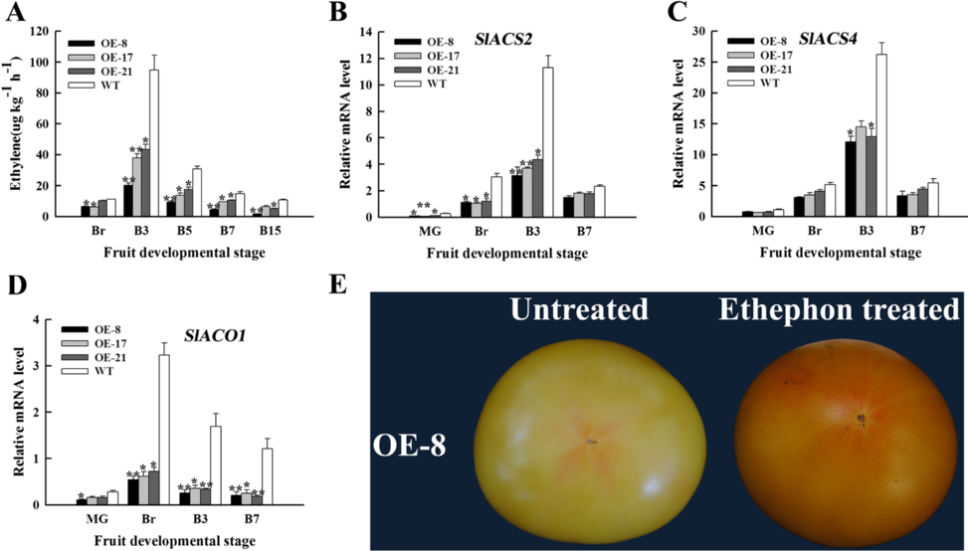
**Ethylene emission and expression of ethylene synthesis genes in OE and WT fruits. (A)** Ethylene production of OE and WT fruits was detected at the indicated stage. **(B) to (D)** qRT-PCR analysis of genes related to ethylene synthesis. The expression of *SlACS2* **(B)**, *SlACS4* **(C)** and *SlACO1* **(D)** were detected between OE and WT fruits. **(E)** Changes in the phenotypes of OE-8 fruits after these fruits were treated with ethephon. MG, mature green; Br, breaker; B3, 3 d after breaker; B7, 7 d after breaker. Data are the means ± SD of three independent experiments. The asterisks indicate statistically significant differences between OE and WT fruits (*P < 0.05, **P < 0.01).

Ethylene regulates carotenoid and lycopene accumulation during ripening by upregulating *SlPSY1* [47]. Fruits released ethylene after Br was measured to determine whether or not the phenotype observed is caused by the change in ethylene contents. Ethylene production of OE and WT fruits had the similar pattern and the climacteric peak of both OE and WT fruits emerged at B3, but the climacteric peaks of the OE fruits were clearly lower than those of the WT fruits; these peaks were reduced by 54% to 79% (Figure 4A). We then detected the relative mRNA levels of the genes related to ethylene biosynthesis. The expressions of *SlACS2*, *SlACS4* and *SlACO1* in the OE fruits exhibited varying degrees of repression compared with those of WT fruits; this result is consistent with the production of ethylene (Figures 4B to 4D). After the fruits of the OE-8 line at the breaker stage were treated with ethephon for 7 d, the phenotypes of the treated fruit could be partly resumed (Figure 4E). These results illustrated that the pigmentation of *SlNAC1* overexpresion tomato fruits is partly dependent on ethylene.

### SlNAC1 can interact with *SlPSY1*, *SlACS2* and *SlACO1* in YIH experiment

**Figure 5:**
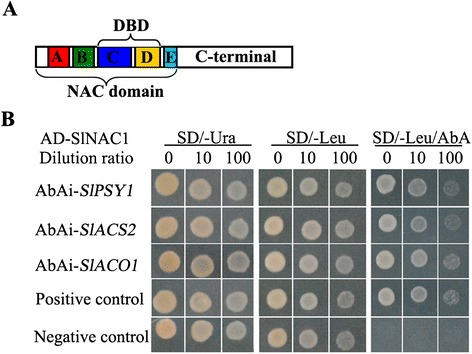
**Yeast one-hybrid assay between SlNAC1 and** ***SlPSY1*** **,** ***SlACS2*** **and** ***SlACO1*** **promoters. (A)** Structure of SlNAC1. The five subdomains (A to E) comprising the NAC domain and the C-terminal are shown. We selected the region from the 65^th^ amino acid to the 149^th^ amino acid containing the DNA binding domain (DBD) to construct the pGADT7 AD-SlNAC1 recombinant plastid. **(B)** Yeast one-hybrid assay results. SD/-Ura, SD medium without Ura; SD/-Leu, SD medium without Leu; SD/-Leu/AbA, SD medium without Leu but containing Aureobasidin A. The p53-AbAi control vector and the pAbAi vector were used as positive and negative controls, respectively.

Transcription factors often regulate gene expression by binding to their promoters to promote or inhibit the corresponding transcription. So we wanted to know whether SlNAC1 could directly bind to the promoters of *SlPSY1*, *SlACS2* and *SlACO1*. The highly conserved positively charged subdomains C and D (Figure 5A) of NAC TFs can bind to DNA [48]. CACG [49] and C/TACG [50] sequences are the core DNA motif recognised by *Arabidopsis* ANAC. Selth et al. [38] reported that the N-terminal 169 amino acid residues of SlNAC1 contain the five conserved subdomains that comprise the NAC domain and SlNAC1 acts as a transcription activator in yeast. On the basis of these previous studies, we selected the fragment containing subdomains C and D of SlNAC1 and the promoter regions containing the C/TACG sequence to perform yeast one-hybrid assay. As shown in Figure 5B, after cotransformation, the yeast strains containing the DNA binding domain of SlNAC1 and the promoters of *SlPSY1*, *SlACS2*, and *SlACO1* could grow on SD/-Ura, SD/-Leu and SD/-Leu/AbA auxotrophic medium, suggesting SlNAC1 might interacted with the regulatory regions of *SlPSY1*, *SlACS2* and *SlACO1* in vivo. This result indicated that SlNAC1 regulates the expression of these genes to finely regulate lycopene and ethylene synthesis.

### Overexpression of *SlNAC1* caused broad ripening changes

**Figure 6:**
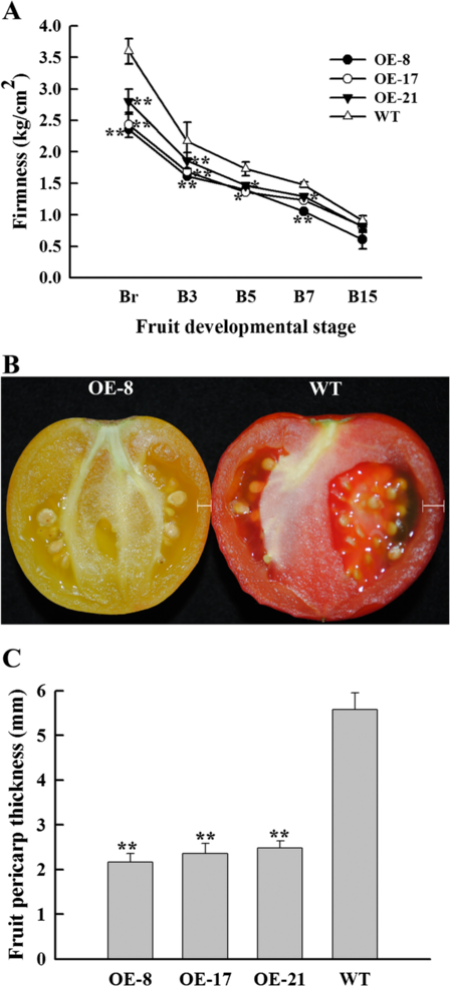
**Fruit firmness and pericarp thickness of OE and WT fruits. (A)** Fruit firmness of OE and WT fruits was evaluated at the indicated stage. Br, breaker; B3, 3 d after breaker; B5, 5 d after breaker; B7, 7 d after breaker; B15, 15 d after breaker. **(B)** Cross-sections of OE-8 and WT fruits at B15. OE fruits showed thinner pericarp (white line indicated) compared with WT fruits. **(C)** The statistics of pericarp thickness between OE and WT fruits in the breaker stage. Data are the means ± SD of at least 10 individual fruits. The asterisks indicate statistically significant differences between OE and WT fruits (*P < 0.05, **P < 0.01).

Fruit softening is associated with ripening and ethylene can accelerate fruit softening. As such, we measured fruit firmness (softening rate) to determine whether or not OE fruits softened later than WT fruits. Our results showed that the firmness of OE fruits was lower than that of the WT fruits at the same stage, meaning the earlier softening of OE fruits, which was inconsistent with ethylene emission (Figure 6A). In addition, fruit pericarp thickness was notably reduced in mature OE fruits (Figure 6B) with over 50% reduction at B15 stage (Figure 6C). Reduced pericarp thickness is correlated with reduced firmness in mature OE fruit, suggesting the contribution of pericarp thickness to fruit softening. Moreover, the seed size of OE fruits was larger than that of WT fruits and OE fruits produced more seeds than WT fruits (data not shown). These results indicated that the overexpression of *SlNAC1* resulted in broad changes of fruit development and ripening.

### Overexpression of *SlNAC1* led to ABA accumulation

**Figure 7:**
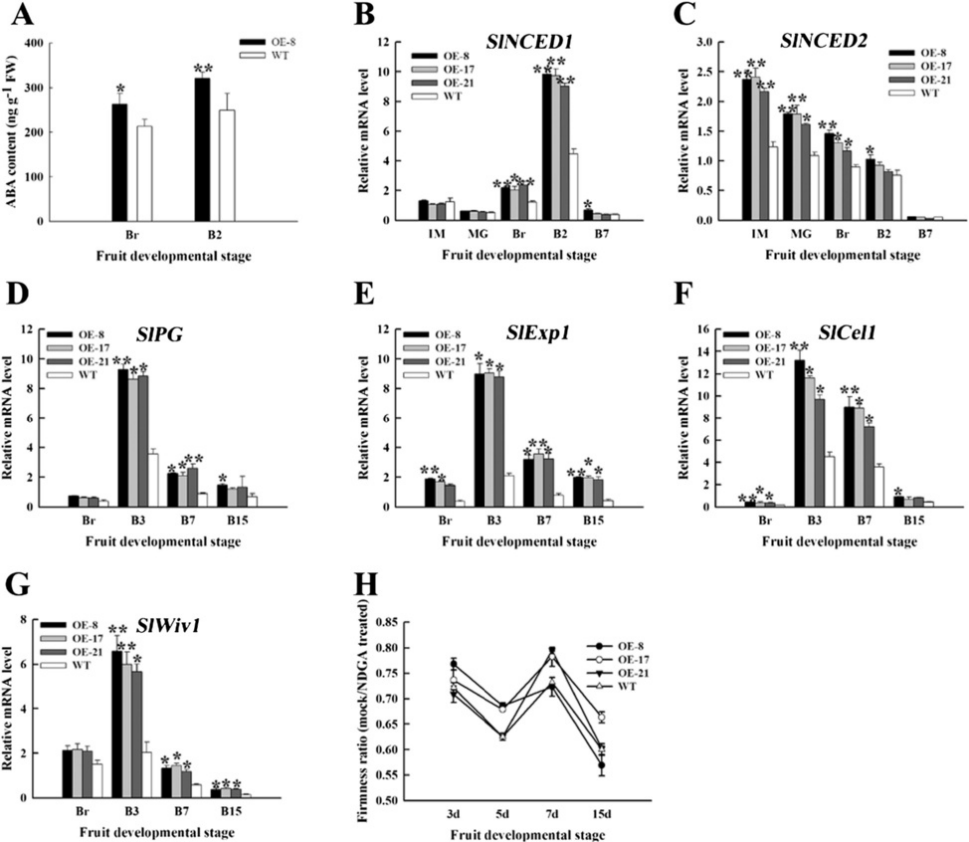
**ABA contents, expression of genes related to ABA synthesis and cell-wall metabolism and NDGA treatment. (A)** ABA contents between OE-8 and WT fruits. **(B)** Expression of *SlNCED1*. **(C)** Expression of *SlNCED2*. **(D)** to **(G)** qRT-PCR analysis of genes related to cell-wall metabolism. The expression of *SlPG* **(D)**, *SlExp1* **(E)**, *SlCel1* **(F)** and *SlWiv1* **(G)** were detected between OE and WT fruits. **(H)** The ratio of firmness of mock fruits to NDGA treated fruits. IM, immature green; McG, mature green; Br, breaker; B2, 2 d after breaker; B3, 3 d after breaker; B7, 7 d after breaker; B15, 15 d after breaker. Data are the means ± SD of three independent experiments. The asterisks indicate statistically significant differences between OE and WT fruits (*P < 0.05, **P < 0.01).

The inconformity between ethylene and fruit softening of OE fruits implied that other factors except ethylene might play roles in fruit softening in tomato. Sun et al. [51] reported that ABA affected cell wall catabolism during tomato fruit ripening by regulating the expression of major catabolic genes and the ABA peak often appears earlier than ethylene peak. To determine whether the different softening rate between OE and WT fruits was resulted from ABA changes, we measured the endogenous ABA contents of OE-8 and WT fruits at Br and B2 stages. Figure 7A showed that ABA contents of OE-8 fruits were higher than those of WT fruits at the same stages. Similarly, the expression of *SlNCED1* (9-cis-epoxycarotenoid dioxygenase) and *SlNCED2* in OE fruits was upregulated compared with those in WT fruits, especially at the early stages of ripening (Figures 7B and 7C). Also, the expression levels of genes related to cell wall metabolism in OE fruits, such as *SlPG*, *SlExp1*, *SlCel1* (endo-1,4-β cellulose) and *SlWiv1* (cell wall invertase), were enhanced compared with those in WT fruits (Figures 7D to 7G). For further validation, OE and WT fruits were treated with NGDA (nordihydroguaiaretic acid, an inhibitor of ABA synthesis) at breaker stage. After NDGA treatment, the firmness of fruits was increased compared with untreated fruits (Figure 7H). These results indicated that overexpression of *SlNAC1* led to ABA accumulation, which at least contributed to fruit softening.

## Discussion

The development of red pigmentation in ripening tomato fruit is caused by the accumulation of lycopene (red) and β-carotene (orange), which represent the majority of the total fruit carotenoids [52]. NOR is a member of NAC transcription factor and its mutant *nor* is phenotypically similar to *rin* in that *nor* fruit fail to produce climacteric ethylene or ripen yet show responsiveness to ethylene at the molecular level while similarly failing to ripen in response to ethylene [53]. NOR RNAi tomato showed ripening defects (slight orange color) from the B + 4 stage in pericarp compared with WT ripening fruits, suggesting alteration of carotenoid composition. Additionally, the placenta of SlNAC4 RNAi fruits failed to acquire red pigmentation and softening appearance from the B + 7 stage [37]. Our results showed that fruits overexpressing *SlNAC1* displayed yellow/orange colour (Figure 1D). The phenotype of *SlNAC1* overexpression fruits was different from that of *nor* and *SlNAC4* RNAi fruits. The yellow/orange fruits were caused by the decreased accumulation of lycopene and elevated lutein and β-carotene (Figure 3B), partially explaining why OE fruits failed to fully turn red. qRT-PCR analysis showed that PSY1, a major regulator of flux towards carotenoid biosynthetic pathway, was significantly reduced because of the overexpression of *SlNAC1* (Figure 3C). In addition, the chromoplast and chloroplast lycopene β-cyclases (CYCB, LCYb and LCYe) in OE fruits were upregulated compared with those in WT fruits (Figures 3D to 3F). The upregulation of *SlPSY1* and the downregulation of *SlCYCB* are at least partly dependent on ethylene; the relative ratio of lycopene to β-carotene in ripening tomato fruit is mediated by both of these processes [43,52,54]. These data suggested that *SlNAC1* participated in tomato fruit pigmentation by regulating the carotenoid biosynthesis pathway in overexpression lines. On the basis of yeast one-hybrid experiment (Figure 5B), we can hypothesise that SlNAC1 may function in the regulation of the carotenoid pathway flux towards lutein and β-carotene and away from lycopene in *SlNAC1* OE fruits.

Tomato is physiologically classified as a climacteric fruit based on the dramatic induction of respiration and ethylene at the onset of ripening. Ethylene functions as a key regulatory hormone in fruit ripening [55]. Ethylene emission of *SlNAC1* overexpression lines was reduced (Figure 4A), suggesting that *SlNAC1* is a negative regulator of ethylene biosynthesis in maturing OE fruit. Ethylene synthesis in ripening tomato fruit is regulated by *ACS* and *ACO* gene families [12]. In tomato, the predominant *ACS* transcripts, namely, *ACS1A*, *ACS2* and *ACS4*, accumulate in ripening fruits. Both *ACS2* and *ACS4* mediate the burst of autocatalytic ethylene synthesis, a process typically observed in climacteric ripening [12]. Oeller et al. [56] found that *ACS2* is the predominant *ACS* mRNA in ripening fruits, and the repression of this gene blocks ripening. The upregulation of *ACS2* [12] and *ACO1* [57] resulted in ethylene and carotenoid accumulation. In agreement with the reduced ethylene production in the OE fruits, the transcript level of crucial genes involved in ethylene synthesis (*SlACS2*, *SlACS4* and *SlACO1*) were suppressed to varying degrees (Figures 4B to 4D). Moreover, exogenous ethephon treatment partially recovered the phenotype of OE fruits (Figure 4E). These results suggested that *SlNAC1* is implicated in OE fruit ripening probably by interacting with ethylene pathway. *TAGL1* positively regulates ethylene synthesis; furthermore, reduced ethylene and a shift toward lutein and β-carotene accumulation occur in ripening fruit as a consequence of the repression of *TAGL1* [36]. The yeast one-hybrid assay results showed that SlNAC1 could bind to the regulatory regions of *SlPSY1*, *SlACS2*, and *SlACO1* (Figure 5B). According to the previous report and the yeast one-hybrid results, we speculated that SlNAC1 negatively regulated ethylene synthesis in OE fruits.

The tomato MADS box TF *RIN*, one of the earliest acting ripening regulators, is required for both ethylene-dependent and ethylene-independent ripening regulatory pathways. *RIN* participates in the regulation of lycopene accumulation and ethylene production by binding to their promoters, such as *ACS2*, *ACS4*, *ACO1* and *PSY1*, which requires *CNR* [22,58,59]. The overexpression of *SlNAC1* and the repression of *RIN* [21], *CNR* [60], *HB-1* [33], *TAGL1* [36] or *SlNAC4* [37] all reduced lycopene accumulation and ethylene synthesis and resulted in similar non-ripening fruits. Furthermore, *RIN*, *CNR*, *HB-1*, *TAGL1* and *SlNAC4* were all reported to be the targets of *RIN* [24-26,37]. Consequently, there might be some relationship between *SlNAC1* and *RIN* in regulating tomato fruit ripening. However, it remains unclear now and will be further investigated.

Softening is another important sign of fruit ripening and related to fruit quality and storage time. The softening of fleshy fruits is caused by changes in the structure and composition of their flesh cell wall. In climacteric fruits, the degradation of pectin and cellulose depends on ethylene during softening [61-63]. The results of fruit firmness analysis showed that the softening rate of OE fruits was inconsistent with ethylene production (Figures 4A and 6A), suggesting there may be an ethylene-independent softening pathway in tomato fruit. It has been reported that *SlNCED1* suppression by RNA interference reduced ABA accumulation in the transgenic fruits, downregulated the genes encoding for major cell wall catabolic enzymes, and then increased the firmness of the transgenic fruits [51]. The levels of ethylene, total carotenoids, lycopene and β-carotene, and the relative transcript levels of *SlACS2*, *SlACS4*, *SlACO1* and *SlPSY1* were enhanced in *SlNCED1* suppression fruit [19]. Overexpressing *SlNAC1* increased the ABA content and expression levels of genes encoding for cell wall metabolism, leading to reduced fruit firmness (Figures 6A and 7). In addition, the levels of ethylene, total carotenoids, lycopene, and the relative transcript levels of *SlACS2*, *SlACS4*, *SlACO1* and *SlPSY1* were reduced in *SlNAC1* overexpression fruit (Figures 3 and 4). These results were inconsist with the previous study. All these changes suggested that ABA negatively regulated fruit firmness. Nevertheless, the detailed mechanism that *SlNAC1* regulates ABA synthesis is unclear now. Vrebalov et al. [36] found that a decrease in the number of pericarp cell layers yield thinner pericarp tissues than normal characteristics. In our study, the pericarp thickness degree of the OE fruits was lower than that of the WT fruits (Figures 6B and 6C). This lower value indicated that the pericarp tissues of the OE fruits contained few cell layers; as such, a thinner pericarp was formed. Saladie´ et al. [64] have demonstrated that fruit turgor is a major determinant of tomato fruit firmness, and as such, direct changes in pericarp thickness combined with resulting effects on water retention are likely to account for the increased softening of *TAGL1* repressed fruit. Thus the reduced firmness or increased softening of *SlNAC1* overexpression fruits might be caused by thinner pericarp. Overall, *SlNAC1* functions in tomato fruit ripening via ethylene-dependent and ABA-dependent pathways in *SlNAC1* overexpression lines.

## Conclusions

*SlNAC1* transcripts accumulated in several tissues, including roots, stems, leaves, flowers, sepals, fruits and seeds, accumulated as fruit ripening occurred and reached the highest level at B7. *SlNAC1* affected tomato fruit pigmentation by regulating the lycopene and ethylene biosynthesis. In addition, *SlNAC1* regulated tomato fruit softening possibly by affecting ABA synthesis and changing the thickness of the pericarp. These data provided a new regulator functioning in fruit ripening and will probably contribute to further mapping of the regulatory network of tomato fruit ripening.

## Methods

### Plant materials and growth conditions

The WT tomato cultivar (*Solanum lycopersium* cv. Zhongshu 6) and T_2_ generation OE lines collected in our greenhouse in May 2012 were grown in a climate-controlled greenhouse at 25°C/18°C at daytime/nighttime under natural light. Different tissues were harvested at designated time. Fruits were harvested in the following stages: immature green (IM); mature green (MG); breaker (Br); and 2, 3, 7 and 15 d after breaker (B2, B3, B7 and B15). Flowers were tagged at anthesis to measure ripening time.

### Generation of transgenic tomato plants

A pair of gene-specific primers (Table 1) was used to clone *SlNAC1* for the construction of overexpressing vector. The fragments were inserted into the expression vector pBI121 at *Bam*HI and *Sal*I sites and then transformed into *Agrobacterium tumefaciens* strain LBA4404. Tomato WT cotyledon explants were transformed as previously described [65].

**Table 1:** **Primers used in this study**

***Primer name***	***Sequence***	***Purpose***
NAC1F	5′-GGAAATGAACAAAGGAGC-3′	Amplification of *SlNAC1*
NAC1R	5′-GTCATGGATCACACTCAA −3′	Amplification of *SlNAC1*
NAC1F’	5′-GGCTTGATGATTGGGTATTGTG-3′	qRT-PCR of *SlNAC1*
NAC1R’	5′-GCTTGTAGTTTCCTTGTTGTCC-3′	qRT-PCR of *SlNAC1*
EF-1αF	5′-GGAACTTGAGAAGGAGCCTAAG-3′	qRT-PCR of *EF-1α*
EF-1αR	5′-CAACACCAACAGCAACAGTCT-3′	qRT-PCR of *EF-1α*
230.2 F	5′-AAGGCTGGACGATTGGGTTCTATG-3′	qRT-PCR of Solyc06g060230.2
230.2R	5′-ATTGCTGCGGCTGAGGATGTG-3′	qRT-PCR of Solyc06g060230.2
410.2 F	5′-TCATCATCGTCATCGTCATCTCAGT-3′	qRT-PCR of Solyc07g063410.2
410.2R	5′-TCCCGCCATAGCAGCCCAAT-3′	qRT-PCR of Solyc07g063410.2
470.1 F	5′-GGCGGTGAGTGAAGGTGATGTAA-3′	qRT-PCR of Solyc11g017470.1
470.1R	5′-GCTGGAATCGGCGTGAAGTT-3′	qRT-PCR of Solyc11g017470.1
620.1 F	5′-GGCAATTCTCGCTGGGCTCAA-3′	qRT-PCR of Solyc12g013620.1
620.1R	5′-GTTGTTGTCGCTGTGAATGTGGTT-3′	qRT-PCR of Solyc12g013620.1
PSY1F	5′-GCATCATATATTACCCCGGCAG-3′	qRT-PCR of *SlPSY1*
PSY1R	5′-TCGGACAAAGCACCATCGA-3′	qRT-PCR of *SlPSY1*
LCYbF	5′-TACCAATGGGTGGTCCACTTC-3′	qRT-PCR of *SlLCYb*
LCYbR	5′-CCTTGCCACCATATAACCGGT-3′	qRT-PCR of *SlLCYb*
LCYeF	5′-ATGGATGTGGCAGGGATTTC-3′	qRT-PCR of *SlLCYe*
LCYeR	5′-CTTTTCTCATGTCATTTGGTGCA-3′	qRT-PCR of *SlLCYe*
CYCBF	5′- GGCTCAATTCGACGTGATCA-3′	qRT-PCR of *SlCYCB*
CYCBR	5′- AGAGTGGTGAAGGGTCAACACA-3′	qRT-PCR of *SlCYCB*
ACS2F	5′-AAGCTTAACGTCTCGCCTGG-3′	qRT-PCR of *SlACS2*
ACS2R	5′-CCACCCTGGCTCTTGACATT-3′	qRT-PCR of *SlACS2*
ACS4F	5′-TCAACGTCTCCCCTGGATCT-3′	qRT-PCR of *SlACS4*
ACS4R	5′-TGCAAGTGCGATCTCCATTG-3′	qRT-PCR of *SlACS4*
ACO1F	5′-TAATGGGAATGGGAAGAAAAGATT-3′	qRT-PCR of *SlACO1*
ACO1R	5′-ACAAAGCAAGATAAAGCACCCC-3′	qRT-PCR of *SlACO1*
DBDF	5′-*GGATCC*ACGGTGAAAAAGAGTGGTA-3′	Cloning the DBD of *SlNAC1*
DBDR	5′-*CTCGAG*AGTTATTGTTCTTGCCAGCAG-3′	Cloning the DBD of *SlNAC1*
PSY1PF	5′-*GGTACC*GGAGTTAGAGGGTAAGTTAC-3′	Cloning the promoter of *SlPSY1*
PSY1PR	5′-*CTCGAG*ACACAGACCATAGCTCTACC-3′	Cloning the promoter of *SlPSY1*
ACS2PF	5′-*GGTACC*CTTTCTCACGTGTAGCTTC-3′	Cloning the promoter of *SlACS2*
ACS2PR	5′-*CTCGAG*TACGCATTAAAAGAAGATCTACG-3′	Cloning the promoter of *SlACS2*
ACO1PF	5′-*GGTACC*CGTGGTCTTTCGAGGTTTGC-3′	Cloning the promoter of *SlACO1*
ACO1PR	5′-*CTCGAG*GACGTAAACATAAGAAATAGC-3′	Cloning the promoter of *SlACO1*
NCED1F	5′-AGGCAACAGTGAAACTTCCATCAAG-3′	qRT-PCR of *SlNCED1*
NCED1R	5′-TCCATTAAAGAGGATATTACCGGGGAC-3′	qRT-PCR of *SlNCED1*
NCED2F	5′-TGGTTTTCATGGGACATTCATTAGC-3′	qRT-PCR of *SlNCED2*
NCED2R	5′-ATCTCCCTTCTCAACTCCCTATTCC-3′	qRT-PCR of *SlNCED2*
PGF	5′-AAGCATGGAATGAAGCATGTTCATCTAG-3′	qRT-PCR of *SlPG*
PGR	5′-CAAAAGCAATCCAAAGCCTTCTATC-3′	qRT-PCR of *SlPG*
Exp1F	5′-AATCAAATGCGGTTTTAACTGGTCAAT-3′	qRT-PCR of *SlExp1*
Exp1R	5′-AATCAAATGCGGTTTTAACTGGTCAAT-3′	qRT-PCR of *SlExp1*
Cel1F	5′-AGTTGCCTCTGAGTTTAGTTGGGATG-3′	qRT-PCR of *SlCel1*
Cel1R	5′-TCCACCTGGGGTTGTCTTAATTTGTA-3′	qRT-PCR of *SlCel1*
Wiv1F	5′-GTGCTGGAGGAAAAACGTGC-3′	qRT-PCR of *SlWiv1*
Wiv1R	5′-GATCGTCTCTGCGCCATTGT-3′	qRT-PCR of *SlWiv1*

The italic indicates restriction sites. GGATCC and CTCGAG represent *Kpn* I and *Xho* I, respectively.

### Carotenoid extraction and high-performance liquid chromatography (HPLC)

Tomato pigments were extracted from the pericarp tissues of fruits at B20 by using the modified protocols of Fraser et al. [66] and Bino et al. [67]. HPLC analysis was performed as described by Verbalov et al. [36]. Frozen tomato powder (0.25 g) was extracted with 1.25 ml of methanol containing 0.1% butylated hydroxytoluene (BHT). The samples were shaken for 5 min, and 1.25 ml of Tris–HCl buffer mixture (pH 7.5, 50 mM) was then added (containing 1 M NaCl). The samples were shaken for 10 min; afterwards, 1 ml of cold chloroform containing 0.1% BHT was added to these samples and then shaken for another 10 min. The samples were subsequently centrifuged at 4°C for 15 min at 4500 rpm. The chloroform phase was collected; the aqueous phase of the samples was then re-extracted with 1 ml of cold chloroform mixture. The chloroform fractions were mixed and dried under N_2_ stream. The dry residue was re-suspended in 1 ml of methyl *t*-butyl ether (MTBE), vortexed, filtered using 0.45 μm and 4 mm polytetrafluoroethylene membrane filter and collected for analysis. All of the solvents used were of HPLC grade. The extracts were kept at 4°C and then shielded from strong light during the entire preparation. An Agilent1200 pump system (Agilent Technologies, USA) with an YMC-Pack reverse-phase C30 column (250 mm × 4.6 mm; 5 μm) was used in compound separation. The mobile phases consisted of acetonitrile:methanol (3:1, v/v) (A) and MTBE (B). Both A and B contained 0.05% triethylamine. The gradient elution process was summarised as follows: B ramped to 55% in 8 min and was maintained in 8 min to 35 min. The column was operated at 30°C with a flow rate of 1 ml min^−1^ and a sample injection volume of 20 μl. The UV spectra were monitored at 450 nm. The experiment was performed using three biological replicates (each with three technical replicates) and gained similar results. Five fruits harvested at different time periods were used as biological replicate.

### Ethylene measurement and ethephon treatment

Fruits were harvested and exposed to air for 3 h to dissipate ethylene released by wounding associated with harvesting. The fruits were sealed in jars and then placed at room temperature for 2 h. An injector was used to mix headspace gas proportionately. Approximately 1 ml of fully mixed headspace gas was injected into a SHIMADZU GC-14C gas chromatograph equipped with a flame ionisation detector. The samples were then compared with a standard gas with known concentration. The measurement was performed by three biological replicates and each replicate contained 10 fruits at least. For ethephon treatment, fruits at the breaker stage were placed in ethephon solution of 3000 μl l^−1^ for 5 min and sealed in jars for another 7 d.

### qRT-PCR

Total RNA was isolated from plant tissues by using an RNAprep plant kit (TIANGEN BIOTECH, http://www.tiangen.com) according to the manufacturer’s protocol. DNase I-treated RNA was reverse transcribed using the M-MLV reverse transcriptase kit (TIANGEN BIOTECH). Real-time PCR was performed using a Bio-Rad CFX96TM real-time PCR system and SYBR real-time Master Mix (TIANGEN). The samples (five fruits collected from different harvests) were represented by three biological replicates (each with three technical replicates); the standard curve method was applied. Template-free, negative and single primer controls were included for each gene analysis. *EF-1α* was used as an internal reference gene to calculate relative transcript levels. The relative gene expression levels were detected using the 2^-ΔΔCT^ method [68]. The primers used for quantitative RT-PCR are listed in Table 1.

### Yeast one-hybrid assay

A Matchmaker Gold yeast one-hybrid library screening system (Clontech, CA, USA) was used to validate the interaction of SlNAC1 and promoters of *SlPSY1*, *SlACS2* and *SlACO1*. The DNA binding domain of SlNAC1 (containing subdomains C and D) was cloned into the pGADT7 vector; the promoter regions (approximately 1000 bp to 1500 bp located upstream of the transcription starting site containing C/TACG sequences) of *SlPSY1*, *SlACS2* and *SlACO1* were cloned into the MCS of pAbAi vector. The transformation of yeast cells and confirmation of positive interactions were performed as described in the Matchmaker Gold Yeast one-hybrid system user manual. The primers used for this experiment are listed in Table 1.

### Fruit firmness measurement

A firmness tester (GY-2) was used to determine fruit firmness as described by Wu and Abbott [69]. A flat probe was placed on the equator of a fruit and used at a displacement rate of 1 mm s^−1^ to press an integrated tomato fruit at a total distance of 3 mm. The maximum force recorded at 3 mm of compression was used as estimated fruit firmness from the averaged value of at least 10 tested fruits and a minimum of three compressions per fruit.

### ABA assay and NDGA treatment of tomato fruits

Samples of tomato fruit were harvested at the proper time and frozen at −80°C. The frozen samples were then ground to powder in liquid nitrogen. The ABA in tomato fruits was extracted and detected as described by Fu et al. [70]. NDGA treatment was performed as described by Zhang et al. [18]. Briefly, tomato fruits at breaker were harvested from the plants and then divided into two groups. 0.5 ml of 100 μM NDGA (group 1) and distilled water (group 2, control) per fruit was injected into the fruits from the pedicle with a medical syringe. Three replications were conducted for each treatment with 10 tomato fruits. The treated fruits were then stored at 20°C and 95% relative humidity (RH) for 3, 5, 7 and 15 d.

### Statistical analysis

Data were presented as mean ± standard deviation. Significant difference between OE lines and WT was analysed using Student’s *t*-test (*P < 0.05, ** P < 0.01).

### Availability of supporting data

Sequence data from this article can be found in the GenBank database (http://www.ncbi.nlm.nih.gov/Genbank) under the following accession numbers: *SlNAC1* (AY498713); *SlPSY1* (EF157835); *SlLCYb* (EF650013); *SlLCYe* (Y14387); *SlCYCB* (AF254793); *SlACS2* (X59139); *SlACS4* (M88487); *SlACO1* (X58273); *SlNCED1* (Z97215); *SlNCED2* (EU912387); *SlPG* (X05656); *SlExp1* (U82123); *SlCel1* (U13054); *SlWiv1* (AB004558) and *EF-1α* (X144491). In addition, the sequences of Solyc06g060230.2, Solyc07g063410.2, Solyc11g017470.1 and Solyc12g013620.1 can be found in PlantTF (http://planttfdb.cbi.pku.edu.cn/) or SGN (http://solgenomics.net/) database.

## Additional file

Additional file 1: Figure S1. The schematic presentation of the carotenoid biosynthesis pathway in plants. DMAPP, dimethylallyl diphosphate; GGPP, geranylgeranyl pyrophosphate; PSY, phytoene synthase; PDS, phytoene desaturase; ZDS, ζ -carotene desaturase; LCYb, lycopene β -cyclase; LCYe, lycopene ε -cyclase; CHYb, β -carotene hydroxylase; ZEP, zeaxanthin epoxidase; VDE, violaxanthin de-epoxidase.

## References

[1] Costa F, Alba R, Schouten H, Soglio V, Gianfranceschi L, Serra S, Musacchi S, Sansavini S, Costa G, Fei ZJ, Giovannoni J (2010). Use of homologous and heterologous gene expression profiling tools to characterize transcription dynamics during apple fruit maturation and ripening. BMC Plant Biol.

[2] Giovannoni JJ (2007). Fruit ripening mutants yield insights into ripening control. Curr Opin Plant Biol.

[3] Moore S, Vrebalov J, Payton P, Giovannoni J (2002). Use of genomics tools to isolate key ripening genes and analyse fruit maturation in tomato. J Exp Bot.

[4] Zouine M, Latché A, Rousseau C, Regad F, Pech JC, Philippot M, Bouzayen M, Delalande C, Frasse P, Schiex T, Noirot C, Bellec A, Klopp C, Berges H, Mariette J, Vautrin S, Causse M, Rothan C (2012). The tomato genome sequence provides insights into fleshy fruit evolution. Nature.

[5] Grierson D, Seymour GB, Giovannoni JJ, Tucker GA, Poole M (2013). Ethylene and the control of fruit ripening. Molecular Biology and Biochemistry of Fruit Ripening.

[6] Bleecker AB, Kende H (2000). Ethylene: a gaseous signal molecule in plants. Annu Rev Cell Dev Biol.

[7] Klee HJ (2002). Control of ethylene-mediated processes in tomato at the level of receptors. J Exp Bot.

[8] Stepanova AN, Ecker JR (2000). Ethylene signaling: from mutants to molecules. Curr Opin Plant Biol.

[9] Wilkinson JQ, Lanahan MB, Yen HC, Giovannoni JJ, Klee HJ (1995). An ethylene-inducible component of signal transduction encoded by never-ripe. Science.

[10] Lincoln JE, Campbell AD, Oetiker J, Rottmann WH, Oeller PW, Shen NF, Theologis A (1993). LE-ACS4, a fruit ripening and wound-induced 1-aminocyclopropane-1-carboxylate synthase gene of tomato (*Lycopersicon esculentum*). expression in *Escherichia coli*, structural characterization, expression characteristics, and phylogenetic analysis. J Biol Chem.

[11] Olson DC, White JA, Edelman L, Harkins RN, Kende H (1991). Differential expression of two genes for 1-aminocyclopropane-1-carboxylate synthase in tomato fruits. Proc Natl Acad Sci U S A.

[12] Barry CS, Llop-Tous MI, Grierson D (2000). The regulation of 1-aminocyclopropane-1-carboxylic acid synthase gene expression during the transition from system-1 to system-2 ethylene synthesis in tomato. Plant Physiol.

[13] Nambara E, Marion-Poll A (2003). ABA action and interactions in seeds. Trends Plant Sci.

[14] Finkelstein RR (2006). Studies of abscisic acid perception finally flower. Plant Cell.

[15] Shinozaki K, Yamaguchi-Shinozaki K (2007). Gene networks involved in drought stress response and tolerance. J Exp Bot.

[16] Galpaz N, Wang Q, Menda N, Zamir D, Hirschberg JP (2008). Abscisic acid deficiency in the tomato mutant *high-pigment 3* leading to increased plastid number and higher fruit lycopene content. Plant J.

[17] Ren J, Chen P, Dai SJ, Li P, Li Q, Ji K, Wang YP, Leng P (2011). Role of abscisic acid and ethylene in sweet cherry fruit maturation: molecular aspects. NZ J Crop Hort.

[18] Zhang M, Yuan B, Leng P (2009). The role of ABA in triggering ethylene biosynthesis and ripening of tomato fruit. J Exp Bot.

[19] Sun L, Yuan B, Zhang M, Wang L, Cui M, Wang Q, Leng P (2012). Fruit-specific RNAi-mediated suppression of *SlNCED1* increases both lycopene and β-carotene contents in tomato fruit. J Exp Bot.

[20] Seymour GB, Ostergaard L, Chapman NH, Knapp S, Martin C (2013). Fruit development and ripening. Annu Rev Plant Biol.

[21] Vrebalov J, Ruezinsky D, Padmanabhan V, White R, Medrano D, Drake R, Schuch W, Giovannoni J (2002). A MADS-box gene necessary for fruit ripening at the tomato *ripening-inhibitor* (*rin*) locus. Science.

[22] Martel C, Vrebalov J, Tafelmeyer P, Giovannoni JJ (2011). The tomato MADS-box transcription factor *RIPENING INHIBITOR* interacts with promoters involved in numerous ripening processes in a *COLORLESS NONRIPENING*-dependent manner. Plant Physiol.

[23] Fujisawa M, Nakano T, Shima Y, Ito Y (2013). A large-scale identification of direct targets of the tomato MADS box transcription factor *RIPENING INHIBITOR* reveals the regulation of fruit ripening. Plant Cell.

[24] Fujisawa M, Nakano T, Ito Y (2011). Identification of potential target genes for the tomato fruit-ripening regulator *RIN* by chromatin immunoprecipitation. BMC Plant Biol.

[25] Fujisawa M, Shima Y, Higuchi N, Nakano T, Koyama Y, Kasumi T, Ito Y (2012). Direct targets of the tomato-ripening regulator *RIN* identified by transcriptome and chromatin immunoprecipitation analyses. Planta.

[26] Qin G, Wang Y, Cao B, Wang W, Tian S (2012). Unraveling the regulatory network of the MADS box transcription factor RIN in fruit ripening. Plant J.

[27] Bemer M, Karlova R, Ballester AR, Tikunov YM, Bovy AG, Wolters-Arts M, Rossetto PB, Angenent GC, Maagd RA (2012). The tomato F*RUITFULL* homologs *TDR4/FUL1* and *MBP7/FUL2* regulate ethylene-independent aspects of fruit ripening. Plant Cell.

[28] Chung MY, Vrebalov J, Alba R, Lee J, McQuinn R, Chung JD, Klein P, Giovannoni J (2010). A tomato (*Solanum lycopersicum*) APETALA2/ERF gene, *SlAP2a,* is a negative regulator of fruit ripening. Plant J.

[29] Dong TT, Hu ZL, Deng L, Wang Y, Zhu MK, Zhang JL, Chen GP (2013). A tomato MADS-box transcription factor, *SlMADS1*, acts as a negative regulator of fruit ripening. Plant Physiol.

[30] Itkin M, Seybold H, Breitel D, Rogachev I, Meir S, Aharoni A (2009). *TOMATO AGAMOUS-LIKE 1* is a component of the fruit ripening regulatory network. Plant J.

[31] Karlova R, Rosin FM, Busscher-Lange J, Parapunova V, Do PT, Fernie AR, Fraser PD, Baxter C, Angenent GC, Maagd RA (2011). Transcriptome and metabolite profiling show that *APETALA2a* is a major regulator of tomato fruit ripening. Plant Cell.

[32] Lee JM, Joung JG, McQuinn R, Chung MY, Fei Z, Tieman D, Klee H, Giovannoni J (2012). Combined transcriptome, genetic diversity and metabolite profiling in tomato fruit reveals that the ethylene response factor *SlERF6* plays an important role in ripening and carotenoid accumulation. Plant J.

[33] Lin Z, Hong Y, Yin M, Li C, Zhang K, Grierson D (2008). A tomato HD-Zip homeobox protein, *LeHB-1*, plays an important role in floral organogenesis and ripening. Plant J.

[34] Pan Y, Bradley G, Pyke K, Ball G, Lu CG, Fray R, Marshall A, Jayasuta S, Baxter C, Wijk R, Boyden L, Cade R, Chapman NH, Fraser PD, Hodgman C, Seymour GB (2013). Network inference analysis identifies an APRR2-Like gene linked to pigment accumulation in tomato and pepper fruits. Plant Physiol.

[35] Shima Y, Kitagawa M, Fujisawa M, Nakano T, Kato H, Kimbara J, Kasumi T, Ito Y (2013). Tomato *FRUITFULL* homologues act in fruit ripening via forming MADS-box transcription factor complexes with *RIN*. Plant Mol Biol.

[36] Vrebalov J, Pan IL, Arroyo AJ, McQuinn R, Chung M, Poole M, Rose J, Seymour G, Grandillo S, Giovannoni J, Irish VF (2009). Fleshy fruit expansion and ripening are regulated by the Tomato SHATTERPROOF gene *TAGL1*. Plant Cell.

[37] Zhu M, Chen G, Zhou S, Tu Y, Wang Y, Dong T, Hu Z (2014). A new tomato NAC (NAM/ATAF1/2/CUC2) transcription factor, *SlNAC4*, functions as a positive regulator of fruit ripening and carotenoid accumulation. Plant Cell Physiol.

[38] Selth LA, Dogra SC, Rasheed MS, Healy H, Randles JW, Rezaian MA (2005). A NAC domain protein interacts with tomato leaf curl virus replication accessory protein and enhances viral replication. Plant Cell.

[39] Ma NN, Zuo YQ, Liang XQ, Yin B, Wang GD, Meng QW (2013). The multiple stress-responsive transcription factor *SlNAC1* improves the chilling tolerance of tomato. Physiol Plant.

[40] Ouyang B, Yang T, Li H, Zhang L, Zhang Y, Zhang J, Fei Z, Ye Z (2007). Identification of early salt stress response genes in tomato root by suppression subtractive hybridization and microarray analysis. J Exp Bot.

[41] Huang W, Miao M, Kud J, Niu X, Ouyang B, Zhang J, Ye Z, Kuhl JC, Liu Y, Xiao F (2013). *SlNAC1*, a stress-related transcription factor, is fine-tuned on both the transcriptional and the post-translational level. New Phytol.

[42] Jin JP, Zhang H, Kong L, Gao G, Luo JC (2014). PlantTFDB 3.0: a portal for the functional and evolutionary study of plant transcription factors. Nucleic Acids Res.

[43] Alba R, Payton P, Fei Z, McQuinn R, Debbie P, Martin GB, Tanksley SD, Giovannoni J (2005). Transcriptome and selected metabolite analyses reveal multiple points of ethylene control during tomato fruit development. Plant Cell.

[44] Burns J, Fraser PD, Bramley PM (2003). Identification and quantification of carotenoids, tocopherols and chlorophylls in commonly consumed fruits and vegetables. Phytochemistry.

[45] Fraser PD, Bramley PM (1994). The purification of phytoene dehydrogenase from Phycomyces blakesleeanus. Biochim Biophys Acta.

[46] Fraser PD, Romer S, Shipton CA, Mills PB, Kiano JW, Misawa N, Drake RG, Schuch W, Bramley PM (2002). Evaluation of transgenic tomato plants expressing an additional phytoene synthase in a fruit-specific manner. Proc Natl Acad Sci U S A.

[47] Maunders MJ, Holdsworth MJ, Slater A, Knapp JE, Bird CR, Schuch W, Grierson D (1987). Ethylene stimulates the accumulation of ripening-related mRNAs in tomatoes. Plant Cell Environ.

[48] Puranik S, Sahu PP, Srivastava PS, Prasad M (2012). NAC proteins: regulation and role in stress tolerance. Trends Plant Sci.

[49] Tran LS, Nakashima K, Sakuma Y, Simpson SD, Fujita Y, Maruyama K, Fujita M, Seki M, Shinozaki K, Yamaquchi-Shinozaki K (2004). Isolation and functional analysis of Arabidopsis stress-inducible NAC transcription factors that bind to a drought responsive cis-element in the early reponsive to dehydration stress 1 promoter. Plant Cell.

[50] Olsen AN, Ernst HA, Leggio LL, Skriver K (2005). DNA-binding specificity and molecular functions of NAC transcription factors. Plant Sci.

[51] Sun L, Sun Y, Zhang M, Wang L, Ren J, Cui M, Wang Y, Ji K, Li P, Li Q, Chen P, Dai S, Duan C, Wu Y, Leng P (2012). Suppression of 9-cis-epoxycarotenoid dioxygenase, which encodes a key enzyme in abscisic acid biosynthesis, alters fruit texture in transgenic tomato. Plant Physiol.

[52] Fraser PD, Truesdale MR, Bird CR, Schuch W, Bramley PM (1994). Carotenoid biosynthesis during tomato fruit development (evidence for tissue-specific gene expression). Plant Physiol.

[53] Lincoln J, Fischer R (1988). Regulation of gene expression by ethylene in wild-type and *rin* tomato (*Lycopersicon esculentum*) fruit. Plant Physiol.

[54] Ronen G, Carmel-Goren L, Zamir D, Hirschberg J (2000). An alternative pathway to beta-carotene formation in plant chromoplasts discovered by map-based cloning of beta and old-gold colour mutations in tomato. Proc Natl Acad Sci U S A.

[55] Barry CS, Giovannoni JJ (2007). Ethylene and fruit ripening. J Plant Growth Regul.

[56] Oeller PW, Lu MW, Taylor LP, Pike DA, Theologis A (1991). Reversible inhibition of tomato fruit senescence by antisense RNA. Science.

[57] Barry CS, Blume B, Bouzayen M, Cooper W, Hamilton AJ, Grierson D (1996). Differential expression of the 1-aminocyclopropane-1-carboxylate oxidase gene family of tomato. Plant J.

[58] Ito Y, Kitagawa M, Ihashi N, Yabe K, Kimbara J, Yasuda J, Ito H, Inakuma T, Hiroi S, Kasumi T (2008). DNA-binding specificity, transcriptional activation potential, and the *rin* mutation effect for the tomato fruit-ripening regulator *RIN*. Plant J.

[59] Kumar R, Sharma MK, Kapoor S, Tyagi AK, Sharma AK (2012). Transcriptome analysis of rin mutant fruit and in silico analysis of promoters of differentially regulated genes provides insight into LeMADS-RIN-regulated ethylene-dependent as well as ethylene-independent aspects of ripening in tomato. Mol Genet Genomics.

[60] Manning K, Tör M, Poole M, Hong Y, Thompson AJ, King GJ, Giovannoni JJ, Seymour GB (2006). A naturally occurring epigenetic mutation in a gene encoding an SBP-box transcription factor inhibits tomato fruit ripening. Nat Genet.

[61] Ergun M, Jeong J, Huber DJ, Cantliffe DJ (2005). Suppression of ripening and softening of ‘Galia’ melons by 1-methylcyclopropene applied at preripe or ripe stages of development. Hort Sci.

[62] Hiwasa K, Kinugasa Y, Amano S, Hashimoto A, Nakno R, Inaba A, Kubo Y (2003). Ethylene is required for both the initiation and progression of softening in pear (*Pyrus communis L.*) fruit. J Exp Bot.

[63] Nishiyama K, Guis M, Rose JK, Kubo Y, Bennett KA, Wangjin L, Kato K, Ushijima K, Nakano R, Inaba A, Bouzayen M, Latche A, Pech JC, Bennett AB (2007). Ethylene regulation of fruit softening and cell wall disassembly in *Charentais melon*. J Exp Bot.

[64] Saladié M, Matas AJ, Isaacson T, Jenks MA, Goodwin SM, Niklas KJ, Xiaolin R, Labavitch JM, Shackel KA, Fernie AR, Lytovchenko A, O’Neill MA, Watkins CB, Rose JK (2007). A reevaluation of the key factors that influence tomato fruit softening and integrity. Plant Physiol.

[65] Yan LH, Zhai QZ, Wei JN, Li SY, Wang B, Huang TT, Du MM, Sun JQ, Kang L, Li CB, Li CY (2013). Role of tomato lipoxygenase D in wound-induced jasmonate biosynthesis and plant immunity to insect herbivores. PLoS Genet.

[66] Fraser PD, Pinto ME, Holloway DE, Bramley PM (2000). Technical advance: application of high-performance liquid chromatography with photodiode array detection to the metabolic profiling of plant isoprenoids. Plant J.

[67] Bino RJ, de Vos CH R, Lieberman M, Hall RD, Bovy A, Jonker HH, Tikunov Y, Lommen A, Moco S, Levin I (2005). The light-hyperresponsive *high pigment*^*-2dg*^ mutation of tomato: alterations in the fruit metabolome. New Phytol.

[68] Livak KJ, Schmittgen TD (2001). Analysis of relative gene expression data using real-time quantitative PCR and the 2^-ΔΔC^T method. Methods.

[69] Wu T, Abbott JA (2002). Firmness and force relaxation characteristics of tomatoes stored intact or as slices. Postharvest Biol Tec.

[70] Fu J, Chu J, Sun X, Wang J, Yan C (2012). Simple, rapid, and simultaneous assay of multiple carboxyl containing phytohormones in wounded tomatoes by UPLC-MS/MS using single SPE purification and isotope dilution. Anal Sci.

