# Supplementary figures and images for: Overexpression of tomato SlNAC1 transcription factor alters fruit pigmentation and softening
Source: BMC Plant Biol. 2014 Dec 10;14:351. doi: 10.1186/s12870-014-0351-y (PMC4272553; doi:10.1186/s12870-014-0351-y)

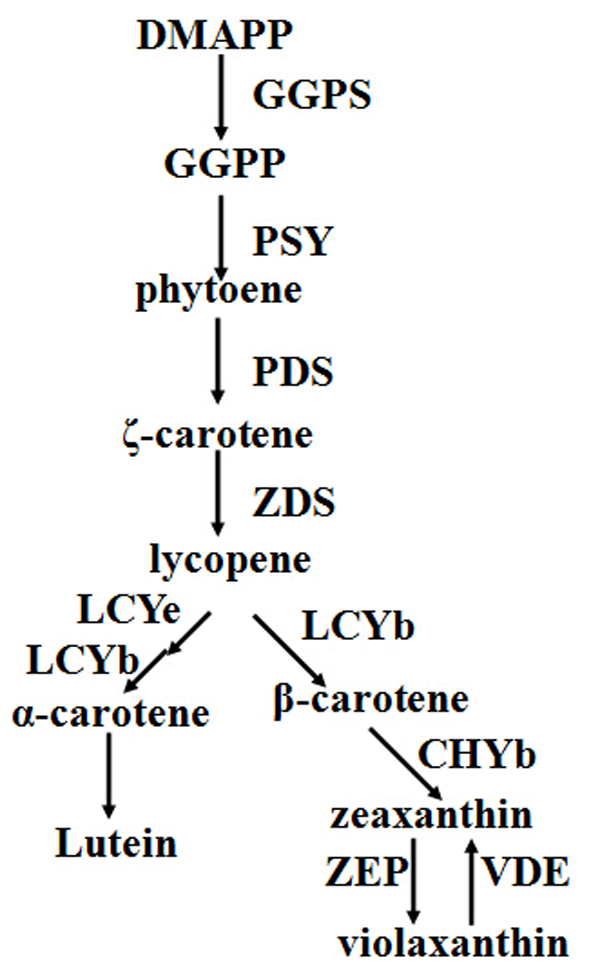

Supplement: Additional file 1: Figure S1. — The schematic presentation of the carotenoid biosynthesis pathway in plants. DMAPP, dimethylallyl diphosphate; GGPP, geranylgeranyl pyrophosphate; PSY, phytoene synthase; PDS, phytoene desaturase; ZDS, ζ -carotene desaturase; LCYb, lycopene β -cyclase; LCYe, lycopene ε -cyclase; CHYb, β -carotene hydroxylase; ZEP, zeaxanthin epoxidase; VDE, violaxanthin de-epoxidase. [file 12870_2014_351_MOESM1_ESM.tiff]
